# CENPA overexpression promotes genome instability in pRb-depleted human cells

**DOI:** 10.1186/1476-4598-8-119

**Published:** 2009-12-10

**Authors:** Angela Amato, Tiziana Schillaci, Laura Lentini, Aldo Di Leonardo

**Affiliations:** 1 Dipartimento di Biologia Cellulare e dello Sviluppo, viale delle Scienze 90128 Palermo, Italy; 2 Centro di OncoBiologia Sperimentale, via San Lorenzo 312, Palermo, Italy; 3 Current address: Division of Medical Oncology, Mayo Clinic, Rochester, MN, USA

## Abstract

**Background:**

Aneuploidy is a hallmark of most human cancers that arises as a consequence of chromosomal instability and it is frequently associated with centrosome amplification. Functional inactivation of the Retinoblastoma protein (pRb) has been indicated as a cause promoting chromosomal instability as well centrosome amplification. However, the underlying molecular mechanism still remains to be clarified.

**Results:**

Here we show that pRb depletion both in wild type and p53 knockout HCT116 cells was associated with the presence of multipolar spindles, anaphase bridges, lagging chromosomes and micronuclei harbouring whole chromosomes. In addition aneuploidy caused by pRb acute loss was not affected by p53 loss.

Quantitative real-time RT-PCR showed that pRB depletion altered expression of genes involved in centrosome duplication, kinetochore assembly and in the Spindle Assembly Checkpoint (SAC). However, despite *MAD2 *up-regulation pRb-depleted cells seemed to have a functional SAC since they arrested in mitosis after treatments with mitotic poisons. Moreover pRb-depleted HCT116 cells showed *BRCA1 *overexpression that seemed responsible for *MAD2 *up-regulation.

Post-transcriptional silencing of *CENPA *by RNA interference, resulting in CENP-A protein levels similar to those present in control cells greatly reduced aneuploid cell numbers in pRb-depleted cells.

**Conclusion:**

Altogether our findings indicate a novel aspect of pRb acute loss that promotes aneuploidy mainly by inducing *CENPA *overexpression that in turn might induce micronuclei by affecting the correct attachment of spindle microtubules to kinetochores.

## Background

Virtually, all solid tumours consist of cells with abnormal chromosomal content known as aneuploidy. Aneuploidy is a form of chromosomal instability (CIN), a condition in which cancer cells lose or gain chromosomes or chromosomal material during mitosis, and experimental evidence strongly suggest that the CIN phenotype may play a role in the onset and/or progression of cancer [1]. Aneuploidy occurrence might generate in a single step multiple genetic changes required both for tumour initiation and progression. However, it is still debated whether aneuploidy is the consequence or the cause of tumorigenesis [2,3]. Molecular mechanisms that ensure accurate chromosome segregation during mitosis are critical for the maintenance of euploidy and errors in this process lead to aneuploidy. Therefore, a major goal is to identify genes that when altered lead to chromosome instability and aneuploidy. Candidate genes encode proteins necessary for correct chromosome transmission, including proteins that function in cell cycle checkpoints, sister chromatid cohesion, kinetochores and at centrosomes [4-6]

Defects of Spindle Assembly Checkpoint (SAC) that prevents chromosome mis-segregation by delaying advance to anaphase until the centromeres of all chromosomes have attached to spindle microtubules, could favour chromosome loss [7]. Altered expression of some SAC components like *Mad2, BubR1, Bub1 and Bub3 *has been also implicated in tumorigenesis [8-11]. However, mutated SAC genes are rarely found in human tumours, thus it is difficult to exclude that this increased expression is simply an indirect consequence of the higher proliferation rate of the tumor.

Faithful transmission of chromosomes relies upon well orchestrated mechanisms including formation of a bipolar spindle and the bi-orientation of mitotic chromosomes to avoid the generation of altered kinetochore attachments, that are considered a cause of lagging chromosomes and micronuclei formation [12]. Thus the correct assembling of the centromere is necessary to ensure the right chromosome segregation. It has been reported that more than one centromere will lead to chromosome breakage or loss, so that one and only one centromere is tolerated per chromosome [7].

Centrosome amplification is a frequent event in several solid tumours [13] as well in leukaemia and lymphoma [14], and it was indicated as a cause of chromosomal instability. Defects in expression of several proteins involved in centrosome duplication/maturation events like Cyclin-E, AurkA or Plk1, could lead to chromosome mis-segregation via multipolar spindle formation because of supernumerary centrosomes [15,16]. Indeed overexpression of these genes was found in different human tumours [17-19].

Functional inactivation of the Retinoblastoma protein (pRb) and p53 has been associated with centrosome amplification, referred to as numerical or structural centrosome dysfunction [20-22] and it was indicated as a cause promoting chromosomal instability [23]. The tumour suppressor pRb is involved in several biological events including synchronization of centrosome duplication and DNA replication. Moreover, it seems that pRb could have a direct role in the assembly of pericentromeric and telomeric heterochromatin domains, though the mechanism remains poorly understood.

Here we show that pRb acute loss induces aneuploidy both in wild type and p53 knockout HCT116 cells. The mechanism by which these cells became aneuploid following pRb depletion involved *CENPA *overexpression. In fact, post-transcriptional silencing of *CENPA *greatly reduced aneuploid cell numbers and micronuclei in pRb depleted cells. Our findings suggest a novel role for pRb in controlling chromosome segregation and in genome stability.

## Results

### Lack of p53 does not affect aneuploidy and centrosome amplification promoted by RB post-transcriptional silencing

**Figure 1:**
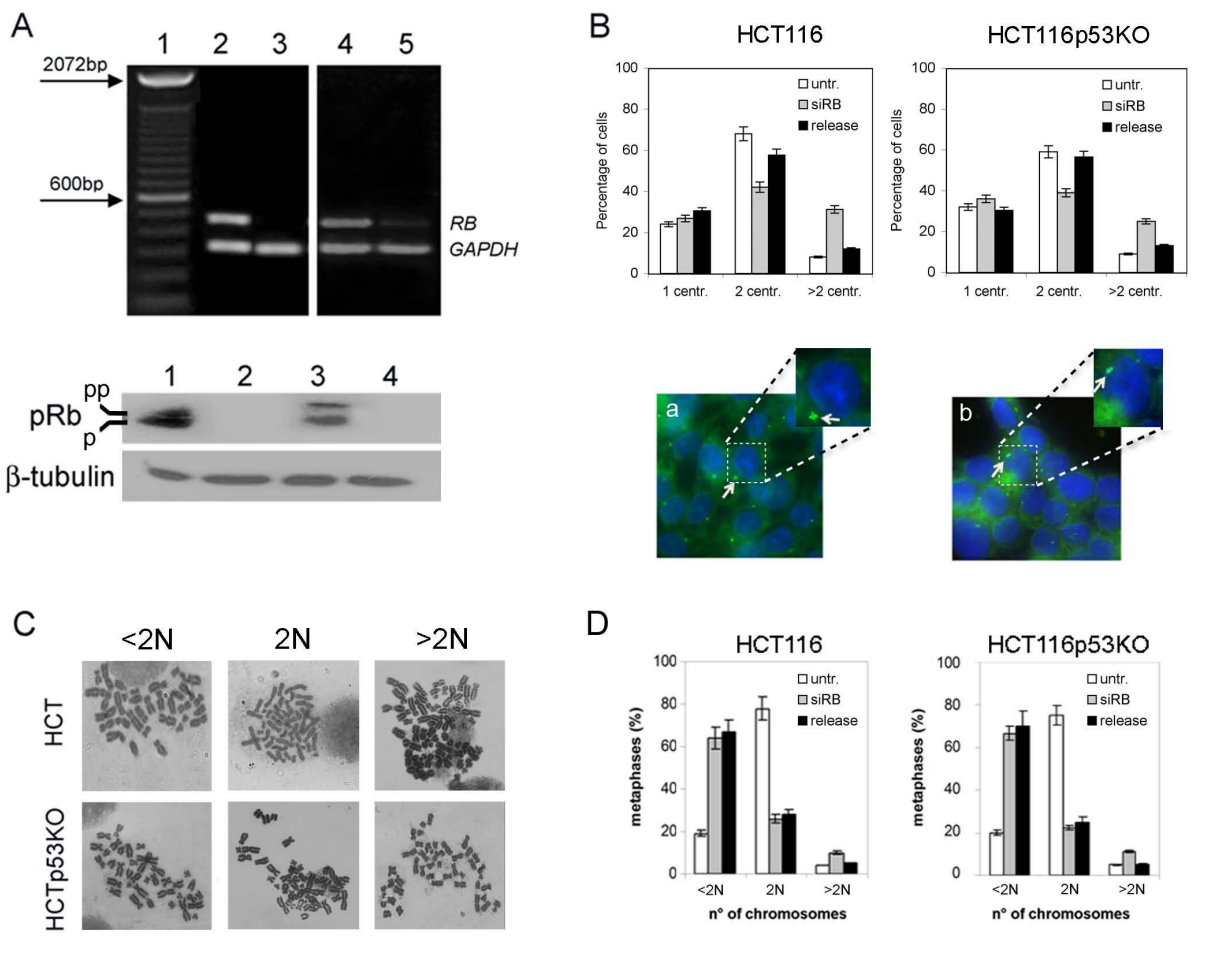
**Supernumerary centrosomes and chromosomal instability promoted by pRb acute loss were not affected by p53 status**. A) RT-PCR (top panel) and Western blot (bottom panel) showing *RB *mRNA and protein levels, respectively, at 72 hours post-transfection in both wild type and p53 knockout HCT116 cells. RT-PCR revealed the presence of *RB *mRNA in both untransfected cell lines, (lane 2: HCT116-wt, lane 4: HCT116p53KO) but not in *RB*-depleted cells (lane 3: HCT116-wt, lane 5: HCT116p53KO). Amplification of GAPDH (330 bp) was used as control of the quality of the cDNA. The 100 bp DNA-ladder was loaded in lane 1 as a size marker. Western blot confirmed selective depletion of pRb in *siRNA-transfected *cells (lane 2: HCT116-wt, lane 4: HCT116p53KO) in comparison with untransfected cells (lane 1: HCT116-wt, lane 3: HCT116p53KO). β-tubulin was used as a loading control. B) Graphs (top panels) showing percentage of cells with 1, 2 or more than 2 (>2) centrosomes in untransfected (untr.), *RB*-depleted (siRB) and released (release) HCT116-wt and HCT116p53KO. Presence of supernumerary centrosomes (bottom panels, white arrow) detected by γ-tubulin immunostaining (green) in both HCT116-wt and HCT116p53KO cells after pRb acute loss (a, b, respectively). A magnification for each cell type is reported on the right. Nuclei were stained with DAPI (blue). C) Representative pictures of diploid (2N), hypodiploid (<2N) and hyperdiploid (>2N) metaphases in both HCT116-wt and HCT116p53KO pRb-depleted cells. D) Graph summarizing the percentages of diploid (2N), hypodiploid (<2N) and hyperdiploid (>2N) metaphases observed in the indicated cell types transfected with siRNA targeting RB (siRb), untransfected (untr.) and released 10 days (release).

Tumor suppressor p53 defects have been associated with centrosome amplification and chromosomal instability in murine fibroblasts [14]. Previously, we demonstrated that alterations of the pRb-E2F pathway induced centrosome amplification and aneuploidy both in human and murine fibroblasts [21]. So it is conceivable that these two mutations might exert a synergistic effect if they act on different pathways that control centrosome homeostasis and aneuploidy. To investigate this issue we used near diploid HCT116 cells both wild-type (HCT116) and p53 knockout (HCT116 p53KO) as a model system. HCT116 and HCT116 p53KO cells were transfected with specific siRNAs to silence *RB *gene expression post-transcriptionally. At 72 hours post-transfection these cells showed very low levels of *RB *transcript (figure 1A, top panel) as determined by RT-PCR analysis. Consistent with this reduction in gene expression Western blot analysis showed decreased levels of phosphorylated and unphosphorylated pRb (figure 1A, bottom panel). We then looked for the presence of supernumerary centrosomes by immunofluorescence microscopy. We found that 35% of HCT116 (figure 1B, left panel) and 25% HCT116-p53KO pRb depleted cells harboured multiple centrosomes (figure 1B, right panel) as detected by staining the centrosome structural protein γ-tubulin (figure 1B, bottom panels). However, after ten days post-transfection, when *RB *gene expression was restored, the number of cells with supernumerary centrosomes decreased in both cell types (figure 1B, release).

Acute loss of pRb increased the number of aneuploid metaphases (figure 1C) up to 70-75% (both <2N and >2N) in comparison to 15% of those observed in control cells, in particular hypodiploid metaphases (<2N) increased up to 60% in both cell types analyzed (figure 1D). At 10 days post-transfection when the pRb level was restored (figure 1D, release) the number of aneuploid metaphases did not decrease, differently of what we observed for the number of cells with supernumerary centrosomes. This last finding suggests that after that aneuploidy has been generated in these cells it was maintained and did not require centrosome amplification anymore.

**Figure 2:**
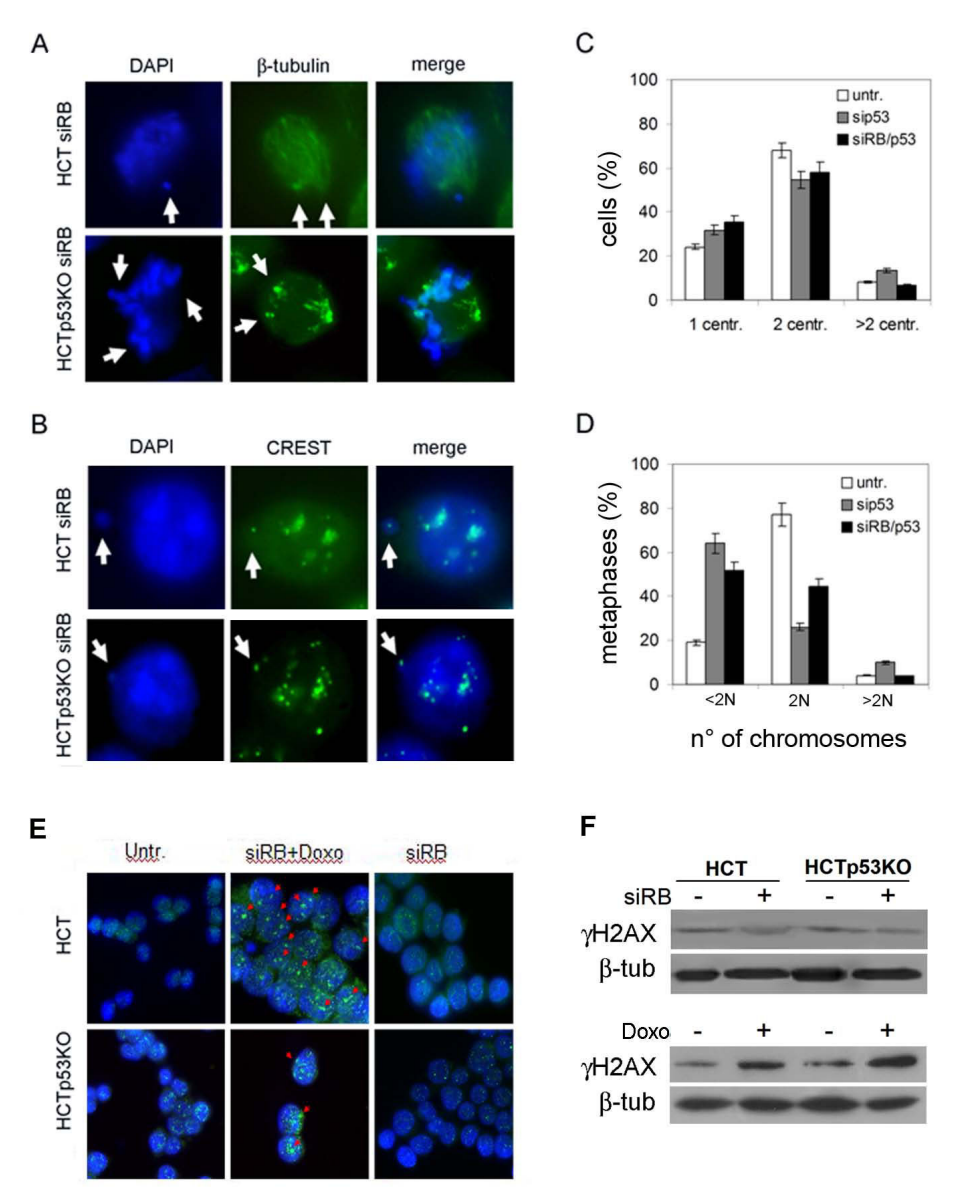
**pRb depleted cells overrode mitosis even when chromosome mis-segregation occurred**. A) Immunodetection of β-tubulin (green) in pRb depleted cells, wild-type (top) and p53-KO (bottom), showing mitotic defects (white arrows). Nuclei were counterstained with DAPI (blue). B) Centromere immunostaining with a CREST antibody detected the presence of centromeres (green) in micronuclei (white arrow) after pRb acute loss in both HCT116 wild-type (top) and p53-KO (bottom) cells. Nuclei were counterstained with DAPI (blue). C) Graph showing the percentage of HCT116 cells with 1, 2 or more than 2 centrosomes at 72 hours post-transfection of siRNAs targeting *p53*, alone or in combination with siRNAs targeting *RB*. D) Graph summarizing the percentages of diploid (2N), hypodiploid (<2N) and hyperdiploid (>2N) metaphases at 72 hours post-transfection of siRNAs targeting *p53*, alone or in combination with siRNAs targeting *RB*. p53 depleted (sip53) and pRb/p53 co-depleted (siRB/p53) cells showed a similar increase in the percentage of hypodiploid metaphases in comparison with untransfected cells. E) Immunofluorescence microscopy detecting γ-H2AX foci (green, red arrows) in wild-type (HCT) and p53 knockout (HCTp53KO) pRb-depleted cells after 24 hours of doxorubicin treatment (siRB+Doxo). In contrast, only background signals were detected in untreated *RB*-depleted (siRB) or untransfected (untr) cells. Nuclei were counterstained with DAPI (blue). F) Western blot analysis did not show increased γ-H2AX levels in HCT and HCTp53KO cells after pRb acute loss (+ siRB) in comparison to untransfected cells (-siRB, top). On the contrary, after doxorubicin (Doxo) treatment γ-H2AX protein levels increased in both cell types (bottom). β-tubulin was used as a loading control.

Altogether the evidence above indicated that lack of p53 did not seem to play any synergistic role with pRb lack, since pRb acute loss triggered multiple centrosomes and aneuploidy at a similar extent in both HCT116 and HCT116-p53KO cells. However, it could be possible that we did not observe any synergistic effect after pRb loss in HCT116-p53KO cells because of the presence of compensatory alterations of the p53 loss, that could have occurred in HCT116-p53KO during selection in culture. We then did co-depletion of pRb and p53 in HCT116 cells by RNAi to determine the immediate effects of simultaneous lack of p53 and pRb. Centrosome analysis by immunofluorescence microscopy as well cytogenetic analysis showed similar results after both simultaneous *RB/p53 *and *p53 *alone post-transcriptional silencing in HCT116 wild-type cells, further supporting results obtained in HCT116 p53KO cells (figure 2C and 2D).

To determine by which mechanism(s) pRb acute loss induced aneuploidy we looked for mitotic defects by immunofluorescence microscopy. This analysis revealed the presence of multipolar mitoses, 20 ± 2% of scored metaphases after pRb depletion, that causing defects in chromosome segregation could in turn result in aneuploid cells (figure 2A).

In addition acute loss of pRb induced micronuclei containing centromeres as revealed by using a specific antibody that detects the kinetochore (figure 2B). After pRb depletion 27 ± 2% of cells showed micronuclei in comparison with only 2-4% of control HCT116-wt cells. By immunofluorescence microscopy we observed the presence of one or more than one centromeric signal in these micronuclei after pRb acute loss, suggesting that they likely contained whole chromosomes and could explain the large number of hypodiploid metaphases observed. Micronuclei could originate other than by defects in chromosome dynamics also by DNA damage. To understand if micronuclei originated because of DNA damage after pRb acute loss in HCT116 cells, we looked at the presence of the histone variant H2AX phosporylation (γ-H2AX) that is considered a marker of DNA damage. To this aim we did immunofluorescence microscopy and immunoblotting in both HCT116 and HCT116-p53KO/pRb -depleted cells. We did not detect γ-H2AX foci in pRb-depleted cells both wild type and p53 knockout as well as in untransfected cells (figure 2E). On the contrary, these analyses showed the presence of (γ-H2AX) foci in pRb-depleted cells treated with doxorubicin (Doxo) that induces DNA breakage (figure 2E, red arrows). These data were confirmed by Western blot analysis showing no increased protein levels for γ-H2AX after pRb acute loss in both cell types (figure 2F, top), in contrast γ-H2AX protein level increased after doxorubicin (Doxo) treatment in both HCT116 cell types (figure 2F bottom).

**Figure 3:**
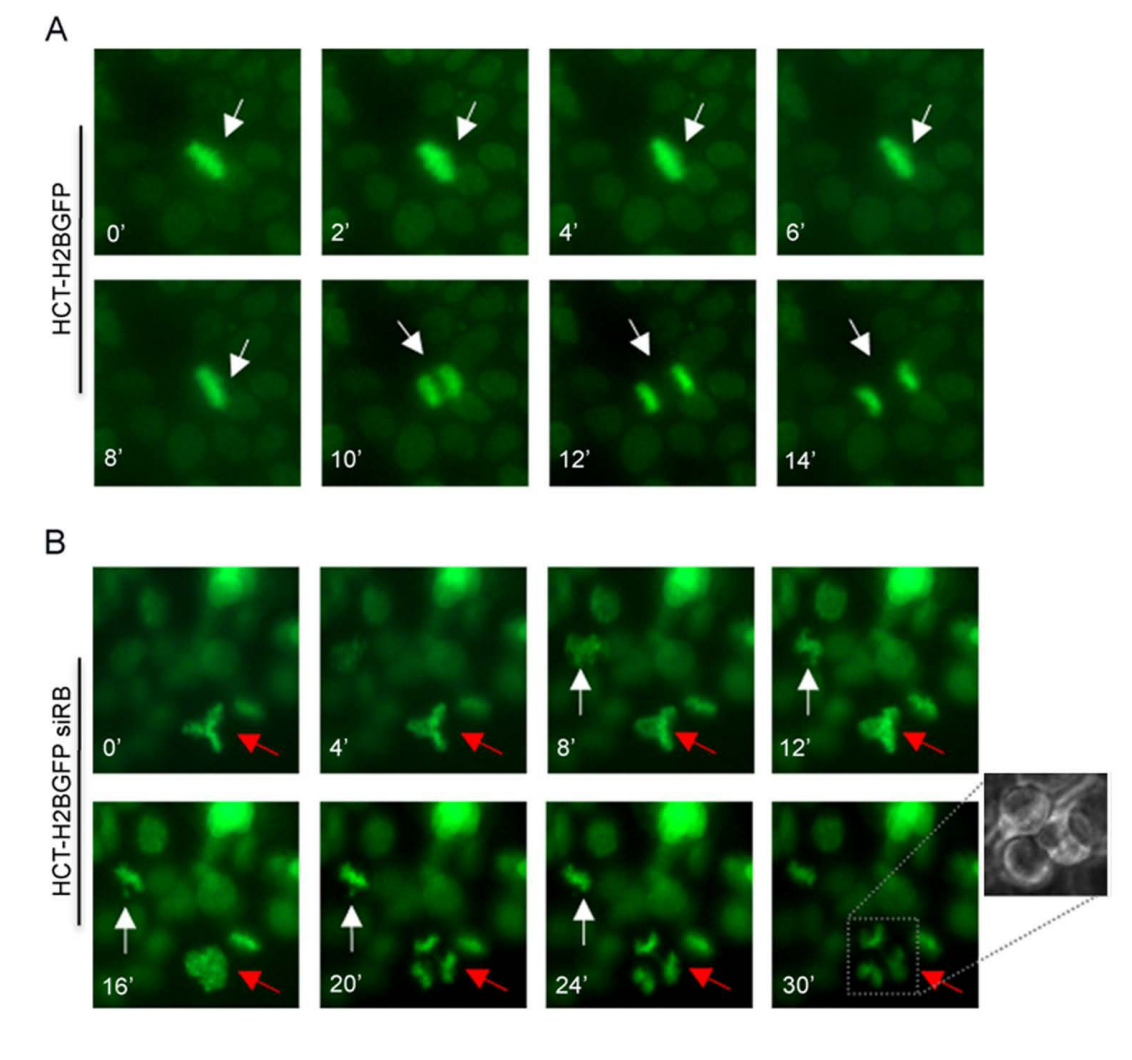
**Chromosome mis-segregation in pRb-depleted cells**. A) Time-lapse video-microscopy done in mitotic HCT116 cells expressing the H2B-GFP gene. Images showing that wild type cells normally progress in mitosis segregating chromosome in two daughter cells. B) RB-depleted cells showing metaphases with unaligned chromosomes that segregate incorrectly generating micronuclei (white arrow) or tripolar metaphases dividing into three daughter cells (red arrow).

Time-lapse video-microscopy experiments done in HCT116 pRb-depleted cells and stably transfected with a plasmid encoding the H2B-GFP protein (figure 3) confirmed that micronuclei and multipolar spindles were generated in these cells. Some pRb-depleted cells exited mitosis despite the presence of tripolar spindles and segregated chromosomes into three daughter cells (figure 3B, red arrow). In some cases, when a bipolar spindle formed, generation of micronuclei was likely responsible for chromosome segregation errors (figure 3B, white arrow).

### pRb acute loss induced altered expression of several centrosome and mitotic genes

**Figure 4:**
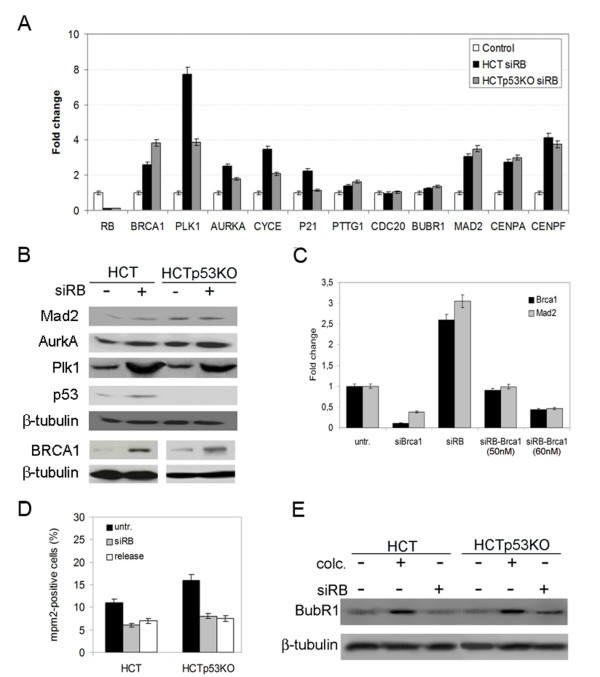
**pRb acute loss induced differential expression of several centrosome and mitotic genes**. A) Real-time RT-PCR showed increased expression levels of genes involved in centrosome duplication (*PLK1, AURKA, and CYCE*), as well in the SAC and mitosis (*BRCA1, PTTG1, CDC20, BUBR1, MAD2, CENPA, and CENPF*) after pRb acute loss in both HCT116wt (HCT) and p53-knockout (HCTp53KO) cells. The x-axis indicates the genes and the y-axis the relative quantification in pRb-depleted cells in respect to the gene expression level in control cells, (untransfected HCT116wt and HCT116p53KO were used as calibrator). B) Western blot showing Mad2, AurkA, Plk1, p53 and BRCA1 protein levels in HCT116 cells both wild-type (HCT, lane 1) and p53-knockout (HCTp53KO, lane 3) and after pRb acute loss (siRB+, lane 2 and lane 4 respectively). β-tubulin was used as a loading control. C) Real-time RT-PCR showing that changes in *MAD2 *transcript depended on *BRCA1 *gene expression levels. *BRCA1 *transcripts were reduced more than 80% at 72 hours post-transfection of siRNAs targeting *BRCA1 *(siBrca1 60 nM). Modulation of *BRCA1 *transcript levels in RB-depleted cells using different siRNA concentration (50 nM and 60 nM) reduced *MAD2 *expression accordingly. D) Graph showing mitotic indices in both wild type and p53-knockout HCT116 cells after pRb acute loss (siRB) and in released cells in comparison with untransfected cells (untr). E) Western blot showing similar BubR1 protein levels in untransfected cells (lanes 1 and 4) and in RB depleted cells (lanes 3 and 6). As expected HCT116 and HCTp53KO cell types after colcemid treatment (lanes 2 and 5) showed increase in BubR1 protein levels.

The Retinoblastoma protein functions primarily as a transcription factor, we then determined if transient loss of pRb affects the expression of genes relevant for the maintenance of genomic stability in HCT116 cells. We looked for changes in gene expression by real-time RT-PCR after *RB *post-transcriptional silencing in both wild type and p53 knockout HCT116 cells. Real-time RT-PCR showed increased expression of *PLK1 *and *AURKA *genes, involved in centrosome regulation, both in wild type and p53 knockout HCT116 cells (figure 4A). Recently, Plk1 has been reported to be regulated by E2F3 transcription factor, a partner of pRb in the regulation of cell cycle transitions, in bladder cancer cells in addition to known E2F3 targets such as Cyclin A [24]. Our recent results suggested that in normal human fibroblasts Plk1 is probably not involved in promoting aneuploidy despite its increased gene expression upon pRb depletion [25]. However, it should be pointed out that cancer cells might behave differently and thus the role of Plk1 overexpression in aneuploidy deserve further consideration.

Among genes working in mitosis and belonging to the Spindle Assembly Checkpoint (SAC) the *BUBR1, CDC20 *and *PTTG*1 genes did not showed marked changes in comparison to control cells. On the contrary pRb acute loss triggered *MAD2 *overexpression in both wild type and p53 knockout HCT116 cells (figure 4A) even though *MAD2 *increased transcript level was not paralleled by the protein level (figure 4B). Recently, *MAD2 *overexpression was reported in stably pRb-depleted human cells that in turn induced chromosomal instability by spindle checkpoint hyperactivation[26]. However, our Western blot experiments did not show increase of Mad2 protein in pRb acutely depleted HCT116 cells, suggesting that differences could exist following transient and stable pRb loss. Likely, stably pRb-depleted cells experienced additional mutations during the selection that altered Mad2 translation and/or stability, making them more permissive to the presence of increased Mad2 protein level. Alternatively, it might be that, as early as 72 h pRb post-transcriptional silencing and/or in the absence of stimuli that should activate the SAC in pRb-depleted cells, increase of *MAD2 *transcription is not promptly followed by its increase at protein level.

It was reported that *BRCA1*, that we found up-regulated in pRb depleted HCT116 cells, could bind and regulate *MAD2 *gene expression [27]. We then wondered if *MAD2 *up-regulation could be correlated with *BRCA1 *overexpression, that we found over-expressed both at transcript and protein levels (figure 4B), in pRb acutely depleted HCT116 cells. To this aim we did simultaneous *RB *and *BRCA1 *post-transcriptional silencing to reduce *BRCA1 *expression to the level found in control cells (basal level) in order to investigate on the existence of a direct relationship between *BRCA1 *and *MAD2 *expression. Quantitative RT-PCR of co-depleted cells showed that *MAD2 *expression levels changed accordingly to the levels of *BRCA1 *transcript. In particular, when *BRCA1 *levels were reduced to the level shown by control cells also *MAD2 *transcriptional levels decreased by 50% (figure 4C). These findings suggest that *MAD2 *up-regulation is the result of *BRCA1 *overexpression that is in turn triggered by pRb depletion.

The presence of mitotic defects in pRb-depleted cells raised the question if the SAC was activated in these cells to halt proliferation of aneuploid cells. To this end we determined the mitotic index (MI) of these cells by fluorescence microscopy using the Mpm2 antibody that detects mitotic phosphorylated proteins. These experiments, carried out in the absence of any mitotic poison, showed that pRb-depleted cells did not increase the MI (figure 4D) as it would be expected after SAC activation. Consistent with this result Mad2 and BubR1 protein levels in pRb-depleted cells were similar to those of control cells (figure 4B, 4E).

### Reduction of CENPA overexpression strongly decrease aneuploidy and micronuclei in pRb depleted cells

**Figure 5:**
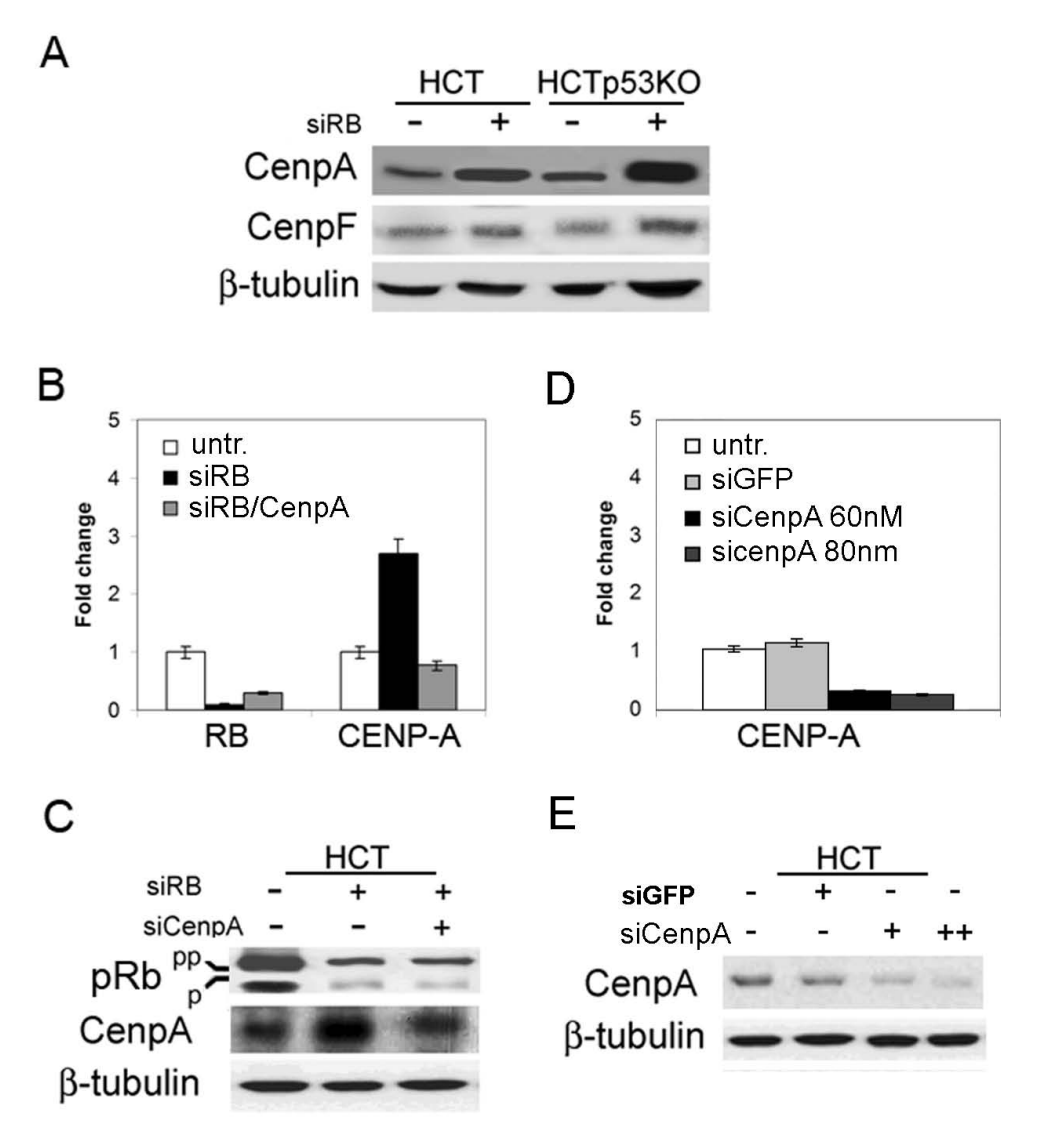
**Reduction of CENPA overexpression by RNAi in pRb depleted cells**. A) Western blot showing CENP-A and CENP-F protein levels in HCT116 cells, wild type (HCT, lane 1), p53-knockout (HCTp53KO, lane 3) and after pRb acute loss (siRB+, lanes 2 and 4), β-tubulin was used as a loading control. B) Real-time RT-PCR showed *CENPA *decreased transcript levels after simultaneous *RB/CENPA *post-transcriptional silencing (siRB/CenpA), in comparison to *CENPA *expression in RB-depleted cells (siRB). RB transcripts were reduced in both pRb- and pRb/CenpA- depleted cells. Untransfected HCT116 cells were used as a calibrator. C) Western blot showing reduction of CENP-A protein levels, in comparison to CENPA expression in RB-depleted cells (siRB+), in cells simultaneously transfected with siRNAs specific for both *RB *and *CENPA *(lane 3). Protein extracts of HCT116 wild type and pRb-depleted cells were loaded in lanes 1 and 2 respectively. β-tubulin was used as a loading control. D) Real-time RT-PCR showing *CENPA *transcript levels in HCT116 cells transfected with a control siRNA targeting GFP (siGFP) and after transfection of siRNAs targeting CENPA (two different doses siCenpA 60 nM, siCenpA 80 nM). E) Western blot showing reduction of CENP-A protein levels after CenpA post-transcriptional silencing (lane 3: 60 nM, lane 4: 80 nM). Normal levels of CENPA were present in untransfected cells (lane 1) and in cells transfected with a control siRNA (lane 2: siGFP).

Real-time RT-PCR showed *CENPA *and *CENPF *overexpression both in wild type and p53-knockout cells after pRb acute loss (figure 4A). However, only *CENPA *overexpression resulted in a sharp protein increase (figure 5A) as showed by Western blotting. Centromere protein A (CENP-A) is a mitotic protein crucial for kinetochore assembly that is necessary for the correct centromere formation and chromosome segregation. *CENPA *overexpression was observed in a high number of aneuploid colon cancers. [28]. Thus, its overexpression could represent a way to trigger genomic instability.

**Figure 6:**
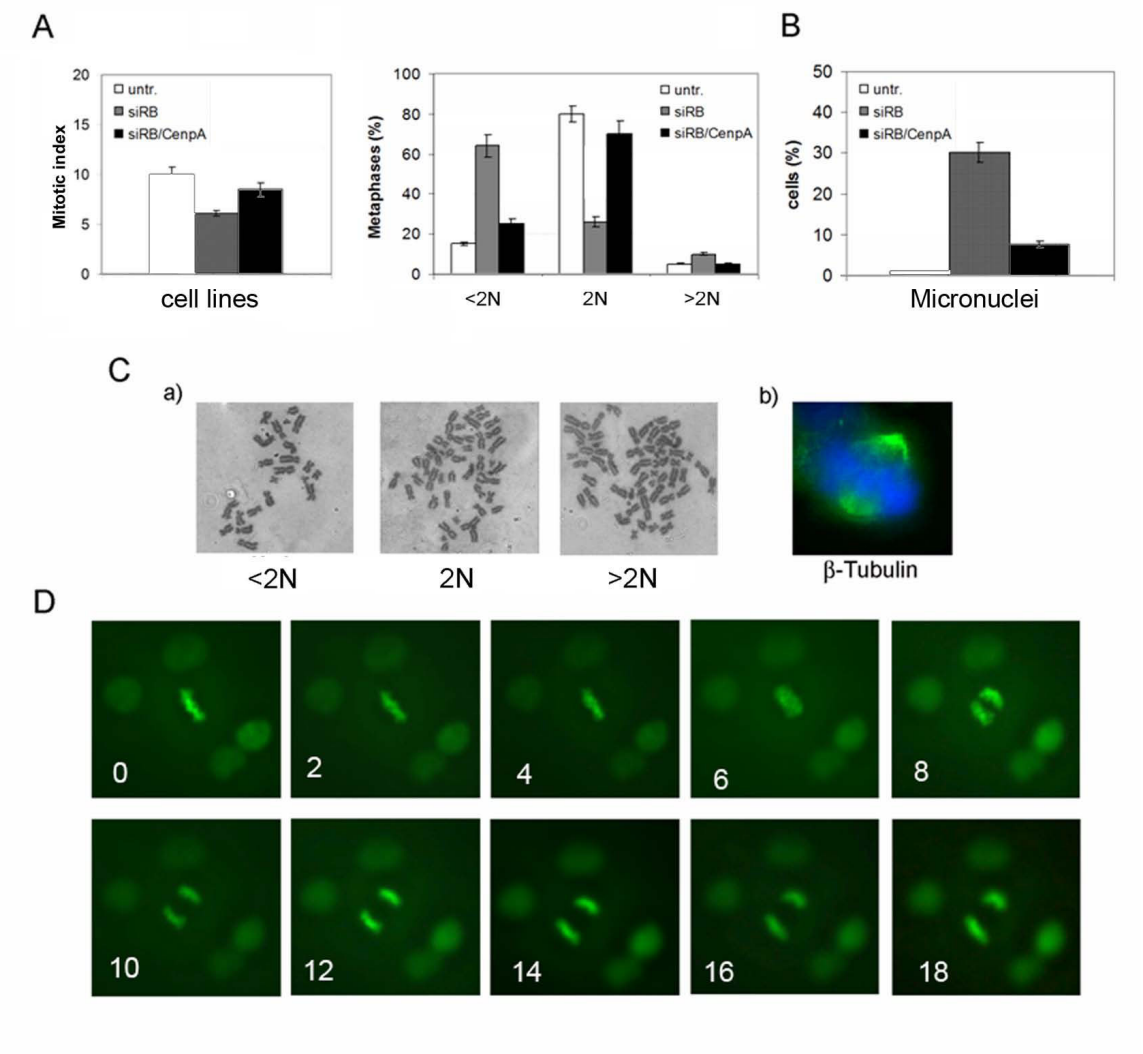
**Inhibition of genomic instability by restoring the correct CenpA protein level in RB-depleted cells**. A) Detection of Mpm2 positive cells by immunofluorescence microscopy in pRb and CenpA co-depleted cells showed a mitotic index (left panel) close to that of wild-type cells. Graph showing (right panel) a large reduction in the percentage of hypodiploid mitosis (<2N) in pRb/CENP-A co-depleted cells (siRB/CenpA) in comparison with pRb depleted cells (siRB). B) Graph showing reduction of cells with micronuclei in pRb/CENP-A co-depleted cells (siRB/CenpA) in comparison with pRb alone depleted cells (siRB). C) **a**- Examples of near diploid (2N), hypodiploid (<2N) and hyperdiploid (>2N) metaphases in pRb/CenpA co-depleted cells; **b**- β-tubulin (green) immunostaining revealed the presence of normal bipolar spindles in pRb/CENP-A co-depleted cells. Chromosomes were counterstained with DAPI (blue). D) Time-lapse video-microscopy of mitotic pRb/CenpA double knockdown cells expressing the H2B-GFP construct showing that they progress normally into mitosis.

To determine whether *CENPA *overexpression observed in pRb-depleted HCT116 cells was involved in genomic instability we investigated if post-transcriptional silencing of *CENPA *by RNAi reduced aneuploidy and micronuclei generation in Rb depleted cells. To this aim we chosen the amount of siRNA duplexes targeting *CENPA *that reduced *CENPA *transcripts to the level resulting in a protein amount similar to that present in untransfected (control) HCT116 cells. Real-time RT-PCR analysis showed 70% and 20% post-transcriptional silencing for *RB *and *CENPA *transcripts respectively (figure 5B), in cells simultaneously transfected with siRNAs targeting *RB *and *CENPA*. Accordingly, the decrease of *RB *and *CENPA *transcripts resulted in low level of pRb and CENP-A proteins close to that present in control cells (figure 5C). CENP-A depleted HCT116 cells showed a decrease of *CENPA *transcript when compared to untransfected cells or cells transfected with a control siRNA (siGFP), that was dependent on the concentration of the siRNA used (figure 5D, and confirmed by Western blot (figure 5E). Co-depletion of pRb and CENP-A did not affect proliferation as suggested by cytofluorimetry (data not shown), and these cells showed a mitotic index close to that of control cells (figure 6A, left panel).

Next we investigated the presence of genomic instability in pRb and CENP-A co-depleted cells. Cytogenetic analyses revealed a sharp decrease of hypodiploid (<2N) mitoses (figure 6A, right panel and 6C, a) as well as of micronuclei exhibiting cells (figure 6B) indicating that restoring the correct *CENPA *expression genomic instability is strongly inhibited. Accordingly, fluorescence microscopy detecting β-tubulin revealed that the majority of these co-depleted cells assembled a normal bipolar spindle (figure 6B, C). In addition we looked at mitosis progression in pRb and CENP-A co-depleted HCT116 cells expressing H2B-GFP by time-lapse video-microscopy. As expected, inhibition of *CENPA *overexpression in *RB*-depleted cell restored the normal mitotic progression (figure 6D).

Altogether these findings strongly suggest that *CENPA *overexpression could be a crucial event that mediates chromosome instability after pRb acute loss.

## Discussion

Defects in chromosome segregation play a critical role in genomic instability and aneuploidy a hallmark of cancer. Indeed gains or losses of chromosomes (i.e., aneuploidy) altering dramatically gene expression could generate in a single step multiple changes required for both tumour initiation and progression. However, the mechanisms implicated in the formation of aneuploid cells are not yet fully understood.

Alterations in genes controlling centrosome homeostasis and defects in genes encoding mitotic checkpoint proteins could trigger aneuploidy. The presence of supernumerary centrosomes by assembling multipolar spindles is considered a possible cause of both chromosomal instability and aneuploidy. Recently, we provided evidence that pRb functional inactivation could drive chromosomal instability by centrosome amplification, referred to as numerical centrosome dysfunction [21]. Also p53 functional inactivation was reported to trigger chromosomal instability by increasing the presence of centrosome amplification in murine cells [14]. Thus, it is possible that these two mutations induce centrosome alterations and aneuploidy by different pathways and in turn might exert a synergistic effect on genomic instability. However, pRb acute loss induced centrosome amplification and aneuploidy at a similar extent in both wild-type and p53 depleted HCT116 cells suggesting that there was not a synergistic effect of these two major pathways in inducing genomic instability.

When analyzing the effects of pRb acute loss at longer times (ten days post-transfection) we observed the reduction of the percentage of cells with supernumerary centrosomes but not that of aneuploid cells. This finding suggests that pRb depletion triggers perpetual genomic instability also in the absence of centrosome alterations. Since the Retinoblastoma protein functions primarily as a transcription factor the observed centrosome defects could be related with the altered expression of genes relevant for centrosome homeostasis, such as *PLK1, AURKA *and *CYCLIN-E*, that we found in pRb depleted HCT116 cells.

Surprisingly, we observed that aneuploid cells induced by pRb depletion were mostly hypodiploid. This unexpected finding is consistent with what has been recently reported for *CENP-E*^+/- ^mice, suggesting that hypodiploid cells are best tolerated than hyperdiploid cells in vivo [29].

By immunofluorescence microscopy we found that micronuclei contained centromeres, suggesting that they could be a means by which pRb-depleted HCT116 cells lost chromosomes. Micronuclei might result from lagging chromosomes that fail to attach to the mitotic spindle in cells that exhibit inactive SAC. However, evaluation of the mitotic index suggested that, when challenged, the SAC functioned correctly in pRb-depleted cells. Alternatively, chromosomes with merotelic orientation, that are not detected by the SAC because they do not expose unattached centromeres, might result in lagging chromosomes that persisting until anaphase onset generate micronuclei and could be lost [12,30]. Consistent with this finding Western blotting did not show Mad2 and BubR1 protein increase, two crucial SAC genes, in these cells. Recently, *MAD2 *up-regulation was reported in stable *RB*-depleted human cells [26] and it was suggested that it could account for chromosomal instability. We found by real-time RT-PCR that *MAD2 *was up-regulated in *RB*-depleted cells, though Western blotting didn't show increase of Mad2 protein. We then wondered if *MAD2 *overexpression could be a side effect of pRb acute loss. Indeed we found that *BRCA1*, a transcription factor able to bind the *MAD2 *promoter [27], was overexpressed in pRb-depleted cells. The finding that reducing *BRCA1 *gene expression, in pRb-depleted cells by RNAi, reduced *MAD2 *gene expression suggests that *MAD2 *overexpression is dependent on *BRCA1 *up-regulation promoted by pRb acute loss.

As an alternative hypothesis, genomic instability in *RB *silenced cells could be the result of micronuclei caused by defects in kinetochore assembling that generated dysfunctional centromere unable to contact correctly mitotic microtubules. Thus, altered expression and dosage of some centromere components, and consequential defects in kinetochore assembling, could trigger micronuclei generation resulting in unfaithful chromosome segregation during mitosis. Indeed, we found altered expression of genes coding for two centromere proteins in acutely pRb-depleted cells. In particular a bioinformatic analysis done on the 5' upstream sequence of the human *CENPA *gene revealed a potential E2F motif (TTTCCCGC) located at -140 from the transcription start site of the *CENPA *gene (A. Di Leonardo, unpublished observation) that could explain the increase of *CENPA *transcript in pRb depleted cells. Alternatively, *CENPA *overexpression could result from up-regulation of some other transcription factors that are under pRb control. The Forkhead box m1 (*FOXM1*) gene that we found up-regulated in pRb depleted human fibroblasts (T. Schilllaci and A. Di Leonardo, unpublished observation) is under the control of pRb and it has been reported to regulate transcription of gene necessary for mitotic progression [31] such as *CENPA*.

Thus, as reported in colorectal cancer tissues [28,32], CENP-A protein overexpression might cause spreading of centromere heterochromatin along chromosome arms and in turn interfere with the correct kinetochore complex assembling and then cause genomic instability.

The sharp decrease of aneuploid metaphases along with micronuclei exhibiting cells observed following pRb and CENP-A co-depletion, strongly suggests that CENPA overexpression causing defects in microtubules-kinetochores anchoring could trigger aneuploidy in pRb-depleted cells.

## Conclusion

Our findings confirm that the Retinoblastoma protein (pRb) plays an important role in controlling chromosome segregation and genome maintenance. Moreover, depletion of pRb promoted chromosome losses during mitosis accounting for aneuploidy regardless the presence of a functional p53 pathway.

We demonstrate that abolishing *CENPA *overexpression in pRb-depleted cells aneuploid metaphases along with micronuclei exhibiting cells decrease greatly. Thus CENPA overexpression that may affect the correct attachment of spindle microtubules to kinetochores, could be considered a major cause of genomic instability.

## Methods

### Cells and Cell Culture

The human colon cancer cell line HCT116 and its derivatives HCT116p53KO (a generous gift of Prof B. Vogelstein) which have both p53 alleles disrupted respectively [33] were cultured in DMEM supplemented with 10% FBS, 100 units/ml penicillin and 0.1 mg/ml streptomycin (Euroclone Ltd UK).

For live cells imaging HCT116 wt and p53KO cells were transfected with the expression vector encoding the H2B histone fused in frame with the Green Fluorescence Protein (H2B-GFP) [34] using the Lipofectamine2000 Reagent (Invitrogen). After 24 hours from transfection cells were selected with 1 μg/mL of blasticidin and stable clones were pooled.

For siRNAs transfection 2 × 10^5 ^cells were plated in 6-well plates and incubated overnight at 37°C. Specific siRNAs duplexes were mixed with Lipofectamine2000 Reagent (Invitrogen), according to manufacturer's recommendation and added to the cells. After 16 h at 37°C, the medium was replaced. To silence RB post-transcriptionally we used a combination of four siRNAs (siRB smart pool Dharmacon, 80 nM) recognizing specific regions of the *RB *transcript, for Brca1 (siBrca1) and Plk1 (siPlk, 60 nM) silencing we used custom single siRNA (MWG). A siRNA duplex targeting the luciferase gene (siLuc, 60 nM, MWG) was used as a control. Analysis of cell cultures was performed after 72 hours from transfection. For proliferation assay cells were plated on 6-well plates in duplicate and at every end point were harvested and stained with Trypan blue solution.

### Western blot analysis

Cells were lysed in SDS/PAGE sample buffer, protein extracts were resuspended in loading buffer (0.125 M Tris-HCl, 4% SDS, 20% v/v Glycerol, 0.2 M dithiothreitol, 0.02% Bromophenol Blue, pH 6.8) and 50 μg of protein (as determined by the Bradford assay) were loaded per lane on a SDS PAGE gel. After gel electrophoresis proteins were electrotransferred onto Immobilon-PVDF membrane (Millipore) blocked in 5% (w/v) no-fat milk in TBST buffer (10 mM Tris pH8.0, 150 mM NaCl, 0.1% Tween 20) at room temperature and incubated overnight at 4°C with the primary antibody. After three washes with TBST buffer the blot was incubated in horseradish peroxidase-conjugated secondary antibody (Santa Cruz Biotechnology, diluted 1:2000) for 1 hour RT. Western blot was probed with mouse monoclonal anti pRB (554136, Becton Dickinson), Plk1 (sc-17783 F-8, Santa Cruz) antibody or p53 (sc-126 DO-1, Santa Cruz) antibody, goat polyclonal Aurora-A (sc-14318 N-20, Santa Cruz), Mad2 (sc-6329 C-19, Santa Cruz) or CenpA (sc-11278 Santa Cruz) antibody, rabbit polyclonal CenpF (sc-22731, Santa Cruz) or H2A.X (Upstate). Equal loading of proteins was evaluated by probing the blot with a mouse monoclonal antibody for β-tubulin (T40-26, Sigma). Blots were developed with chemiluminescent reagent (SuperSignalWest Pico, Pierce Rockford, IL) and exposed to CL-Xposure film (Pierce Rockford, IL) for 1 to 5 min.

### Cytogenetic analysis

Two days after transfection 4 × 10^4 ^cells were seeded on rounded glass coverslips. And incubated with 0,2 μg/ml Colcemid (Sigma, demecolcine) for 2 hours, resuspended in warm hypotonic buffer (75 mM KCl), allowed to swell for 10-15 minutes at 37°C, fixed by addition of cold methanol/acetic acid (3:1 v/v). The slides were air-dried and stained with 3% Giemsa in phosphate-buffered saline for 10 min. Chromosome numbers were evaluated using a Zeiss Axioskop microscope under a 100× objective. At least 50 metaphases were analyzed at each time point.

### Immunofluorescence microscopy

γ-tubulin detection: To detect centrosomes, 4 × 10^4 ^cells were grown on glass coverslips, fixed in methanol at -20°C, permeabilized with 0.01% Triton X (Sigma) and blocked with 0.1% BSA both at room temperature. Then, coverslips were incubated with a mouse monoclonal antibody against γ-tubulin (Sigma, diluted 1:250 in PBS-BSA 0,1%) overnight at 4°C, washed in PBS and incubated with a FITC-conjugated goat anti-mouse (Sigma, diluted 1:100 in PBS-BSA 0,1%) for 1 hour at 37°C.

β-tubulin detection: To detect centrosomes, 4 × 10^4 ^cells were grown on glass coverslips, fixed in 3.7% formaldehyde solution for 10 minutes at 37°C, then permeabilized with 0.01% Triton X (Sigma) and blocked with 0.1% BSA both at room temperature. Then, coverslips were incubated with a mouse monoclonal antibody against β-tubulin (Sigma, diluted 1:200 in PBS-BSA 0,1%) overnight at 4°C, washed in PBS and incubated with a FITC-conjugated goat anti-mouse (Sigma, diluted 1:100 in PBS-BSA 0,1%) for 1 hour at 37°C.

Mitotic Index Evaluation and phosphorylated H2A.X immunostaining: HCT116 cells, both wild type and p53-knockout, were grown on glass coverslips after siRNAs transfection. For phosphorylated H2A.X immunostaining one coverslip per cell type was incubated with 2 μg/ml Doxorubicin (Doxo) for 7 hours in order to have a positive control. Coverslips were fixed with methanol at -20°C, permeabilized with 0.01% Triton X (Sigma, St. Louis, MO) and blocked with 0.1% BSA, both at room temperature. Then, coverslips were incubated with a mpm2 mouse monoclonal antibody (5 μg/ml in PBS-BSA 0.1%, Upstate) or with phosphorylated H2A.X rabbit polyclonal antibody (1:100 in PBS-BSA 0.1%, Upstate) overnight at 4°C, washed in PBS and incubated with a FITC-conjugated goat or rabbit anti-mouse (Sigma, diluted 1:100 in PBS-BSA 0,1%) for 1 hour at 37°C.

Centromere detection: To detect centromeres, 4 × 10^4 ^cells were grown on glass coverslips, fixed 5 minutes in methanol/DMEM 1:1 (v/v) and then 30 minutes in methanol 100% on ice. Coverslips were washed with wash solution (0,1% BSA in PBS 1×) at room temperature and then incubated with a human anti-nuclear antibody overnight at 4°C (Antibodies Inc, Davis, USA), washed and incubated with a FITC-conjugated anti-human antibody (Antibodies Inc, Davis, USA), for 1 hour at 37°C. For each immunofluorescence analysis described above nuclei were visualized with 4', 6-Diamidino-2-phenylindole (DAPI) and cells examined on a Zeiss Axioskop microscope equipped for fluorescence. Images were captured with a CCD digital camera (Axiocam, Zeiss) and then transferred to Adobe Photoshop for printing.

### Total RNA preparation and RT-PCR

Total RNA was extracted from untransfected or RB knockdown HCT116 cells, both wild type and p53-knockout, by RNAeasy Mini kit according to the manufacture's instruction (Qiagen). Total RNA (200 ng) was used for reverse transcriptase reaction (RT) which was carried out using the One step RT-PCR kit (Qiagen), RT was performed for 30 minutes at 50°C and 15 minutes at 95°C. The Amplification cycle (94°C for 45 sec., 55°C for 45 s, and 72°C for 1 min) was repeated 30 times. PCR primers for RB and GAPDH were designed to produce a DNA fragment of 440 bp or 330 bp in length, respectively. The sequences of primers used were: 5'-CAGGGTTGTGAAATTGGATCA-3' and 5'-GGTCCTTCTCGGTCCTTTGATTGTT-3' for RB; 5'-TGACATCAAGAAGGTGGTGA-3' and 5'-TCCACCACCCTGTTGCTGTA-3' for GAPDH.

### Real-Time RT-PCR

A subset of genes was selected for quantitative reverse transcription RT-PCR. Primers were designed with Primer Express software (Applied Biosystems) choosing amplicons of approximately 70-100 bp crossing an exon/exon boundary to minimize the chance that a signal was from contaminating DNA. Total RNA of each sample was retro-transcribed in cDNA by High Capacity cDNA Archive kit (Applied Biosystems) for 10 minutes at 25°C and 2 hours at 37°C Two microliters (60 ng) of cDNA produced from reverse transcription reactions was added to 12.5 μl of SYBR green 2× PCR Master Mix (Applied Biosystems), 1.88 μl of 2 μM specific primer pair, and dH2O up to 25 μl final volume. A 7300 Real-Time PCR systems was used to perform real-time fluorescence detection during PCR made up of a step for AmpliTaq Gold Enzyme activation at 95°C for 10 minutes followed by 40 cycles of PCR (95°C for 15 sec and 60°C for 60°C for 1 minute). Data were analyzed by averaging triplicate Ct (cycle threshold). Glyceraldehyde-3-phosphate dehydrogenase was used as internal control. Following the PCR reaction, a melting curve assay was performed to determine the purity of the amplified product. The sequences of primers used were: GAPDH: 5'-CTCATGACCACAGTCCATGCC-3' and 5'-GCCATCCACAGTCTTCTGGGT-3', 

AURKA: 5'-TTTTGTAGGTCTCTTGGTATGTG-3' and 5'GCTGGAGAGCTTAAAATTGCAG-3',

PLK1: 5'-CGGAAATATTTAAGGAGGGTGA-3' and 5'-GGACTATTCGGACAAGTACG-3',

MAD2L1: 5'-CTCATTCGGCATCAACAGCA-3' and 5'-TCAGATGGATATATGCCACGCT-3',

RB: 5'-GCAGTATGCTTCCACCAGGC-3' and 5'-AAGGGCTTCGAGGAATGTGAG-3'

BRCA1: 5'-CCTTGGCACAGGTGTCCAC-3' and 5'-GCCATTGTCCTCTGTCCAGG-3',

PTTG1: 5'-CGGCTGTTAAGACCTGCAATAATC-3' and 5'-TTCAGCCCATCCTTAGCAACC-3'

CDC20: 5'-GAGGGTGGCTGGGTTCCTCT-3' and 5'-CAGATGCGAATGTGTCGATCA-3',

BUB1B: 5'-TACACTGGAAATGACCCTCTGGAT-3' and 5'-TATAATATCGTTTTTCTCCTTGTAGTGCT-3'

CENPA: 5'-GATTCTGCGATGCTGTCTGG-3' and 5'-CAGGCCTTTGGAACGGTGTT-3'

CENPF: 5'-TCTGCCTTGAAGAAGAACTCTCA-3' and 5'-TGAATGCAATGGAGACACTCACT-3'

CYCE: 5'-CTCCAGGAAGAGGAAGGCAA-3' and 5'-ATTATTGTCCCAAGGCTGGC-3'

### Live cell time-lapse imaging

Cells were imaged in T25 flasks in CO_2_-independent medium (GIBCO-BRL) at 37°C. Chromosome segregation was monitored on a heating plate (37°C) with an inverted microscope and a 40× objective on a LEICA inverted microscope equipped with a mercury 100 W lamp. Under UV excitation, cells in metaphase were identified and mitotic progression was monitored every 3 minutes on average. Mitotic progression was documented with a CCD digital camera (AxioCam Zeiss) and then transferred to Adobe Photoshop.

## Competing interests

The authors declare that they have no competing interests.

## Authors' contributions

AA carried out siRNA experiments, real-time RT-PCR, immunofluorescence microscopy, Western blotting, participated in the design of the study and helped to draft the manuscript. TS did cytogenetic and time-lapse experiments, immunofluorescence microscopy, and helped to draft the manuscript. LL carried out RNA preparation for real-time RT-PCR, participated in western blotting and helped to draft the manuscript. ADL conceived of the study, participated in its design and coordination, and wrote the manuscript. All authors read and approved the final manuscript.
